# A Case of Laceration of the Uterus

**Published:** 1859-11

**Authors:** L. Beecher Todd

**Affiliations:** Lexington, Kentucky


### ART. III.—*A Case of Laceration of the Uterus.* By L. BEECHER TODD, M.D., of Lexington, Kentucky.

I WAS requested, on Thursday afternoon, March 4th, 1858, about two o'clock, to visit a married lady, forty years of age, who, as her husband informed me, was taken in her tenth labor about daylight. On entering her chamber, I found her stretched on a mattress before the fire, white, and cold as marble, almost pulseless, and apparently lifeless. "What does this mean?" I inquired of the midwife whom I found in attendance, at the same time administering hot stimulants, and ordering her to be placed very carefully on her bed, when I received the following graphic history of her case:—

"Her labor-pains awoke her about five o'clock this morning; pulse good; courage fair; temperature of skin excellent all morning. About ten o'clock her pains became not only more frequent, but more severe, and so continued until about noon, when, having discharged a large quantity of liquor amnii, she had several very severe pains, followed by a terrible bearing-down effort, when she screamed out that '*her child was turning over and over, and had gone up;*' immediately after which every pain ceased entirely, perfect exhaustion and languor ensued, and they thought best then to call a physician."

She had been in this condition for two hours, and did not appear to suffer much; but complained of pressure in her bowels and fullness about her womb; disposed to stupor, though frequently restless. A per-vaginam examination revealed the entire vaginal canal and uterus, as far as I could introduce my index and middle fingers, filled with a large quantity of coagulated blood, which I proceeded to remove as rapidly as possible. Failing, however, to touch the child—the hemorrhage still continuing—I ordered stimulants and cold applications over the uterine region; and wishing to go to a drug-store on the street, I met my friend Dr. Joseph S. Halstead, and requested him to visit the case with me. Her condition, pulse, sensation, and appearance, were about as when I first saw her. Upon a thorough vaginal examination and about the umbilical region, we both came immediately to the painful and terrible conclusion that the child was thrust into the cavity of the abdomen—the *womb was lacerated.*

Professor J. M. Bush and Dr. Bruce saw the case, who concurred in the distressing and fatal prognosis. We remained with her until six o'clock, alleviating, as far as possible, her sufferings, which seemed to increase, concluding that, in her present prostrated condition, no *operation* could be thought of.

About seven o'clock I returned with my friend Dr. E. L. Dudley. During this visit, with his assistance, I was enabled for the first time to effect a thorough examination of the extent of the laceration, position of child, and general condition of the parts. Introducing my entire hand into the uterus, and passing my index and middle fingers through the laceration, which seemed to involve the greater part of fundus, at least six or eight inches in length, I distinctly felt the child, which caused her intense pain, lying crosswise in the abdominal cavity, with the head in the left iliac fossa. It is needless to mention that all efforts to move or return the child proved in vain. Ordered perfect quiet, morphia, and hot applications. Saw her about midnight. Pulse scarcely perceptible; extremities cold; sinking gradually; morphia repeated.

Friday morning, five o'clock.—Found her, after a painful and restless night, rapidly sinking. She remained during the night perfectly conscious of her hopeless condition, which she bore with Christian fortitude, until about seven o'clock, when she expired.

An autopsy was made six hours after death, in the presence of Professors Bush and Skillman and Dr. Bruce. The abdominal cavity was found completely filled with coagulated blood. The child, a male, was unusually large, and thrust, with the placenta and membranes, crosswise in the abdominal cavity, the head lying in the left iliac fossa; the fundus of the uterus was lacerated to the extent of eight or ten inches, and the womb was somewhat contracted and filled with coagula.

